# Modeling test learning and dual-task dissociations

**DOI:** 10.3758/s13423-020-01761-4

**Published:** 2020-06-15

**Authors:** Tobias Johansson

**Affiliations:** Department of Psychology, Kristianstad University, 291 88 Kristianstad, Sweden

**Keywords:** Dual task, Memory, Exemplar model, Test learning

## Abstract

Much of cognitive psychology is premised on the distinction between automatic and intentional processes, but the distinction often remains vague in practice and alternative explanations are often not followed through. For example, Hendricks, Conway and Kellogg (*Journal of Experimental Psychology: Learning, Memory, and Cognition*, *39*, 491–1500, 2013) found that dual tasks at training versus at test dissociated performance in two different artificial grammar learning tasks. This was taken as evidence for underlying automatic and intentional processes. In this article, a different explanation is considered based on test learning and similarity, where participants are assumed to update their knowledge at test. Contrasting formal memory models of test learning are implemented, and it is concluded that the models account for the relevant dissociations without assuming a distinction between automatic and intentional processes.

## Introduction

The distinction between automatic and intentional processes is commonplace in psychology. Typically, in contrast to intentional processes, automatic processes are described as being applied effortlessly with relatively little need for cognitive resources (Hasher & Zacks, 1979). Therefore, if a task is performed well even when cognitive resources are strained, we may want to declare the process behind performance as automatic. An illustrative example of this reasoning can be found in a study by Hendricks, Conway, and Kellogg (2013), who concluded that the effects of a dual task on implicit learning provided evidence for automatic and intentional processes. In this article, I consider a different explanation of their results partly based on on-line test learning. To this end, I extend a formal memory model to incorporate dual-task influences.

The kind of implicit learning studied by Hendricks et al. (2013) is called artificial grammar learning (AGL). Typically, there are two phases, a training phase and a test phase. In the training phase participants memorize letter sequences that follow a set of rules (a grammar). These rules involve sequential constraints, specifying which letters may follow other letters in specific positions. The participants are not informed about the existence of underlying rules. After the training phase, participants are asked to classify new sequences, half of which follow the rules (grammatical) and half of which violate the rules (ungrammatical). Typically, participants perform above chance even though they are unable to articulate the rules. In so-called *transfer* experiments, the letter set is changed between training and test. Even in this case, participants have been demonstrated to perform above chance.

Hendricks et al. (2013) used a dual task methodology so that some participants performed a dual task - i.e. two simultaneous tasks - at training (memorization of sequences plus a digit span task) or at test (classification of sequences plus a digit span task). Hendricks et al. (2013) found that for standard AGL (with unchanged letters between training and testing) a dual task *at test* disrupted performance so that participants could not distinguish between grammatical and ungrammatical sequences, but a dual task *at training* did not disrupt performance. In contrast, for transfer AGL a dual task disrupted performance regardless of whether the dual task occurred at training or at test. From this, Hendricks et al. (2013) concluded that transfer AGL requires intentional learning processes, while standard AGL does not, because participants were able to learn standard AGL despite performing dual tasks at training.

Translating the results of Hendricks et al. (2013) into a difference between automatic and intentional processes may be intuitively appealing. However, a well-known fact is that dissociations between tasks do not imply different underlying processes (Dunn & Kirsner, 2003). The AGL literature itself is rich with examples where dissociations have been accounted for by models assuming a single underlying process. For example, Kinder and Shanks (2001) used a single-system simple recurrent network in order to simulate a dissociation between classification and recognition due to amnesia, without assuming separate processes behind classification and recognition. Jamieson, Holmes, and Mewhort (2010) experimentally demonstrated the same kind of dissociation in healthy participants by manipulating study time, and then simulated the results using a single-system memory model, namely Minerva II (Hintzman, 1984). Tunney and Shanks (2003) used a single-system simple recurrent network to simulate automatic and controlled influences in AGL in a design where participants are trained on two grammars and then asked to endorse test items from either only one of the grammars or from both (see Higham, Vokey, & Pritchard, 2000). The simulations showed that manipulating response parameters of a single-system model operating only on the similarity constraints embodied in the stimulus materials was enough to reproduce dissociations between automatic and controlled influences (see also Vokey & Higham, 2004). In addition, there are many examples outside the AGL framework using the same type of modeling approach (e.g., Curtis & Jamieson, 2019; Kinder & Shanks, 2003; Nosofsky & Zaki, 1998). The current work fits in to this tradition of using computational models to account for dissociations, rather than assuming that dissociations directly reflect underlying processes.

An assumption implicit in both Hendricks et al. (2013) and most formal models of AGL is that no learning occurs during test. The test phase is merely assumed to involve expression of acquired knowledge. Simply storing items at test may induce performance benefits if the test items are classified on the basis of similarity to not only training items, but also previously encountered test items. The possibility of test learning has been recognized for a long time in AGL research, especially with respect to the question of how to establish that learning has occurred (Reber & Perruchet, 2003). Different forms of test learning have been demonstrated in AGL for trained groups (Beesley, Wills, & le Pelley, 2010), untrained control groups (Redington & Chater, 1996), and computational models (Rohrmeier & Cross, 2014). In addition, test learning has been demonstrated in many other areas of cognitive psychology, including prototype-learning of dot patterns (Palmeri & Flanery, 1999; Zaki & Nosofsky, 2007) and learning curves with respect to the testing effect (Roediger & Smith, 2012).

In this article I aim to show that the pattern of results observed by Hendricks et al. (2013) may be a result of test learning rather than a dissociation between automatic and intentional processes. The basic assumptions are that participants learn at test and that a dual task reduces learning. The similarity constraints of test items may enable participants to distinguish between grammatical and ungrammatical items using information from the test phase, without feedback (Beesley, Wills, & le Pelley, 2010). The extent to which information in the test phase helps distinguish between grammatical and ungrammatical test items will depend on the exact information contained in the test items. So, observing that a dual task at training leaves performance unaffected does not necessarily imply automatic learning. Participants may classify correctly despite a dual task at training, because they learn useful information during test.

## Holographic exemplar model and test learning

In order to model test learning I consider the holographic exemplar memory model (HEM) proposed by Jamieson and Mewhort (2011), which in turn is an extension of a model (Minerva II) proposed by Hintzman (1984). HEM uses holographic representation of sequences. This is done by applying non-commutative circular convolution recursively in order to represent structure beyond that of single-letter units. Suppose the sequence MVXT is encoded as M, V, and XT. Each of these three components is then represented by separate vectors and the vectors are then summed. The bigram XT is formed by convolution of X and T, so that the representation of the sequence becomes M +V + X*T, where * denotes non-commutative circular convolution. Jamieson and Mewhort (2011) describe this and other model operations in more detail.

Sequence encoding is governed by a learning rate parameter *L*, indicating the probability with which features of a vector are stored correctly. With probability 1-*L*, features are set to 0. Once encoded the sequences are part of the memory store. A sequence presented at test activates all sequences in memory as a non-linear function of similarity. This produces an echo, a constructed representation reflecting the memory activation produced by the test sequence. The similarity between the echo and the test sequence is denoted the intensity and is used for classification. If the intensity exceeds a decision criterion *k* the sequence is classified as grammatical.

A simple way to incorporate test learning is to extend HEM to encode test sequences to the memory store as they are presented throughout testing. In order to model dual tasks at training and test, I use separate learning rate parameters for training and test sequences and denote these *L*_*train*_ and *L*_*test*_, respectively. Dual tasks are assumed to strain cognitive resources and correspond to lower learning rates.

The model parameters are shown in Table 1. There are six parameters in total: two learning-rate parameters (*L*_*train*_ and *L*_*test*_) that take either high or low values depending on condition (single vs. dual task)*,* the decision criterion (*k*), the dimensionality of the component vectors (*n*), the highest level of encoded sequential structure (*q*), and the number of encoded subcomponents of a sequence (*g*). The *g* parameter reflects the fact that participants may not encode all possible subcomponents of a sequence. For example, presented with MVXTXVT, a participant may perhaps only encode a small number of subcomponents, say MV and XTX. In the simulations *g* random components are selected from each presented sequence. The *q* parameter reflects the fact that participants may only encode sequential structure up to a certain level. For example, if *q* = 3, participants are assumed to encode up to three-letter subcomponents (letters, bigrams, and trigrams).

**Table 1:** Model parameters, values, and their meaning

Parameter	Value	Meaning
L_train_	Dual task: {.1, .2, .3} Single task: {.8, .9, 1}	Probability of encoding feature correctly at training
*L*_*test*_	Dual task: {.1, .2, .3} Single task: {.8, .9, 1}	Probability of encoding feature correctly at test
*g*	{2, 3, 4, 5}	Number of sampled components of a sequence
*q*	2 (bigrams only)	Encoded *q*-gram structure (1 = letters, 2 = bigrams, etc.)
*k*	Set to match endorsement rates	Decision criterion
*n*	100	Dimensionality of letter vectors

## Modeling dual tasks

A dual task at test could have the effect of reducing learning rate at test, but it could also quite plausibly have other effects. Here, I present a set of three different extended HEM models aimed to tease apart these factors to some extent.

The first model includes test learning and degrades a test item by the test learning rate before classification. Here, a dual task at test affects both test learning and classification. The second model includes test learning but degrades the test item only after classification. Thus, the second model assumes that a dual task at test only affects test learning, not classification. The third model does not include test learning, but degrades the test item before classification. This model enables an assessment of performance when a dual task is assumed to affect classification without test learning. I refer to these three models as HEM-TC, HEM-T, and HEM-C, respectively, where T indicates that the model involves Test learning and C indicates that a test item presented for Classification is degraded by the test learning rate parameter.

In order to model the results of Hendricks et al. (2013), I treated the mean difference between the hit- and false-alarm rates as the outcome variable. A hit means saying “grammatical” to a grammatical item and a false alarm means saying “grammatical” to an ungrammatical item. In accordance with Jamieson and Hauri (2012), each subcomponent vector consisted of *n =* 100 random normal deviates with mean 0 and standard deviation 1/*n*. The *g* and *q* parameters should plausibly be constrained to reflect quite limited sequential structure when applied to AGL (Jamieson & Mewhort, 2011). In these simulations the *g* parameter was allowed to vary from 2 to 5. I initially explored the *q* parameter from 1 to 3, but as single-letter knowledge is often not a diagnostic predictor in AGL and only seemed to add random noise to the model predictions, I implemented the models without single-letter knowledge. Here I report results for *q* = 2 (bigrams only) as higher values produced far too erratic and noisy results. Bigram knowledge is often a central component of AGL (Perruchet & Pacteau, 1990), but ultimately the kind of knowledge acquired depends on encoding (Jamieson, Vokey, & Mewhort, 2017).

Each condition in the experiments of HCK involved a dual task at training, dual task at test, dual task at both training and test, or no dual tasks. In the models, these conditions correspond to high or low learning rates at training and/or test. A dual task is assumed to correspond to a lower learning rate. Through simulation I explored a coarsely specified parameter space to obtain an equally coarse multidimensional likelihood function for each of the models (cf. Rohrmeier & Cross, 2014). These likelihoods were then averaged in order to obtain a composite likelihood for each model. This corresponds to a Bayesian marginal likelihood with a uniform prior over the model parameters. The ratio of the likelihoods of two models further corresponds to the Bayes factor, indicating the relative support for one model over the other across the parameter space. In addition to Bayes factors, I also report maximum likelihood ratios. In the latter case, only the highest likelihood of a model is considered, providing a basis for how well a model and its best parameter combination can predict the data, instead of model performance on average across the parameter space.

The parameter space consisted of all combinations of *g* = {2, 3, 4, 5} and three parameter values for each of the learning rates *L*_*train*_ and *L*_*test*_ (where the values depend on condition so that dual task = {.1, .2, .3 and single task = {.8, .9, 1}). This results in 36 different combinations for each model and each of the two simulated experiments. The standard and transfer AGL experiments were simulated independently from each other, but the conditions within each were not.

For each combination I simulated 100 iterations of 20 participants in each of the four conditions (high/low learning rate at training/test). The number of participants in Hendricks et al. (2013) varied from 16 to 26 depending on experimental condition. The likelihood is the probability of the observed empirical data given the parameter values of the model. I consider a model to have reproduced the observed data on a given iteration if the root mean square error *E* for the model with respect to the observed mean differences between hits and false alarms for the four conditions does not exceed *E* = 0.02. This criterion is further motivated in the *Results and discussion* section.

I also conduct a form of posterior predictive checks for each model, both by using the maximum likelihood parameter estimates and by sampling from the posterior distribution of model parameters, and checking the extent to which the implied model results match the observed data from HCK. The posterior distribution used here is based on uniform priors over the parameters and is thus identical to the simulated multidimensional likelihood function. Put simply, likelihood ratios such as Bayes factors indicate which model is least bad, while posterior predictive checks indicate whether the models are any good at all.

The decision criterion *k* was set to produce the proportions of “grammatical” responses observed by HCK (see their Table 2). Thus, *k* took different constrained values depending on simulated condition. Classification was simulated in retrospect, so that simulation of a participant resulted in an intensity distribution with one intensity value for each test item. The decision criterion *k* was then set to the appropriate percentile of the intensity distribution in order to produce a desired proportion of “grammatical” responses. As noted, when simulating standard AGL (same letter set for training and testing), I implemented the models to take only bigrams into account (*q* = 2), including starting and end positions of a sequence (cf. Servan-Schreiber & Anderson, 1990). For example, the sequence XXVT would first be represented as sXXVTe, where s and e stand for “start” and “end,” respectively. The sequence was then parsed into its constituent bigrams: sX, XX, XV, VT, and Te. Then, a random sample of *g* of these bigrams were convolved to represent the sequence, retaining an expected proportion of *L* of its features and setting the rest to 0 before encoding it to memory. Sometimes a sequence could have fewer than *g* components. In that case, sampling with replacement was allowed, and otherwise sampling was done without replacement.

**Table 2:** Bayes factors (BFs) and maximum likelihood ratios (MLRs) for standard and transfer artificial grammar learning (AGL)

	Standard AGL		Transfer AGL	
	BF	MLR	BF	MLR
HEM-TC/HEM-T	4.37	1.67	-	-
HEM-TC/HEM-C	-	-	1.30	1.24
HEM-T/HEM-C	-	-	-	-

*Note*: Empty cells involve model comparisons including models with zero or very close to zero likelihood across the parameter space. These models are not considered further

For transfer AGL, the representational scheme for standard AGL does not work, because the letter set is changed between training and testing so that the expected similarity between training and testing sequences is zero. In transfer AGL, participants have been shown to rely on repetition information (Lotz & Kinder, 2006), especially local repetition information (see Brooks & Vokey, 1991, for a related account). Representations of local repetition capture the immediate repetitions in a sequence. For example, the sequence XXVT can be represented as sABBe, where A = repetition (XX) and B = non-repetition (XV, and VT). When simulating transfer I implemented the models with this representational scheme, both for training and test sequences. The *q* parameter was set to 1 in order to reflect bigram knowledge, because a single letter in the repetition scheme (A or B) reflects a bigram (repetition or non-repetition).

A possible assumption motivating this representational scheme for transfer AGL is that participants directly encode the repetition patterns of the sequences. If so, utilization of repetition knowledge would not require additional inferential processes. The current simulations explore the viability of such a framework. The assumption of direct repetition encoding is not necessarily correct of course. For example, it could be that participants infer a mapping between training and test sequences at test (Dienes, Altmann, & Gao, 1999). If so, dual tasks could be expected to interfere with these inferential processes in line with HCK. My aim here though, was to explore the implications of not assuming these inferential processes. Matlab model code is available for download at https://osf.io/awpgm/.

## Results and discussion

The root mean square error criterion *E* for counting something as reproducing the observed data is somewhat arbitrary. It needs to be relatively small in order to stay close to the data, but it also needs to be large enough to respect the resolution of the model outputs. I initially tried *E* = 0.01, which resulted in zero likelihood for all models. I then tried *E* = 0.02, and these are the results I report here. Under these conditions HEM-C could not account for standard AGL and HEM-T could not account for transfer AGL. For these two cases I also tried higher values of *E* up to 0.05 and also investigated posterior predictive checks without being able to reproduce the observed data patterns. These two cases are therefore not included in the following comparisons.

**Fig. 1:**
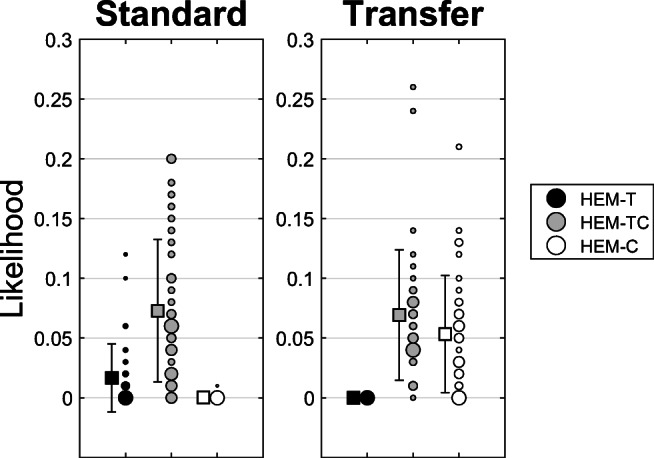
Simulated likelihood values for each model and experiment. Likelihood is here the probability of generating the empirical means of hits minus false alarms in the four experimental conditions within a root mean square error window of no more than 0.02, given a specific combination of model parameters. Squares show means ± 1 standard deviation. Circles show simulated values with size proportional to frequency. For the HEM-T model in the transfer condition all values were zero and for the HEM-C model in the standard condition all values but one (.01) were zero

For standard AGL the HEM-TC model has the highest probability of generating the observed data across the parameter space, while for transfer AGL the HEM-TC and HEM-C model are quite close to each other. The Bayes factors and maximum likelihood ratios in Table 2 quantify these results. For standard AGL, the HEM-TC is about four times more likely to produce the data across the parameter space compared to the HEM-T model, but the maximum likelihoods are relatively close for the two models. For transfer AGL, the HEM-TC and HEM-C models are more or less on equal footing with respect to both Bayes factor and maximum likelihood ratio. The likelihood distributions are shown in Fig. 1.

**Fig. 2:**
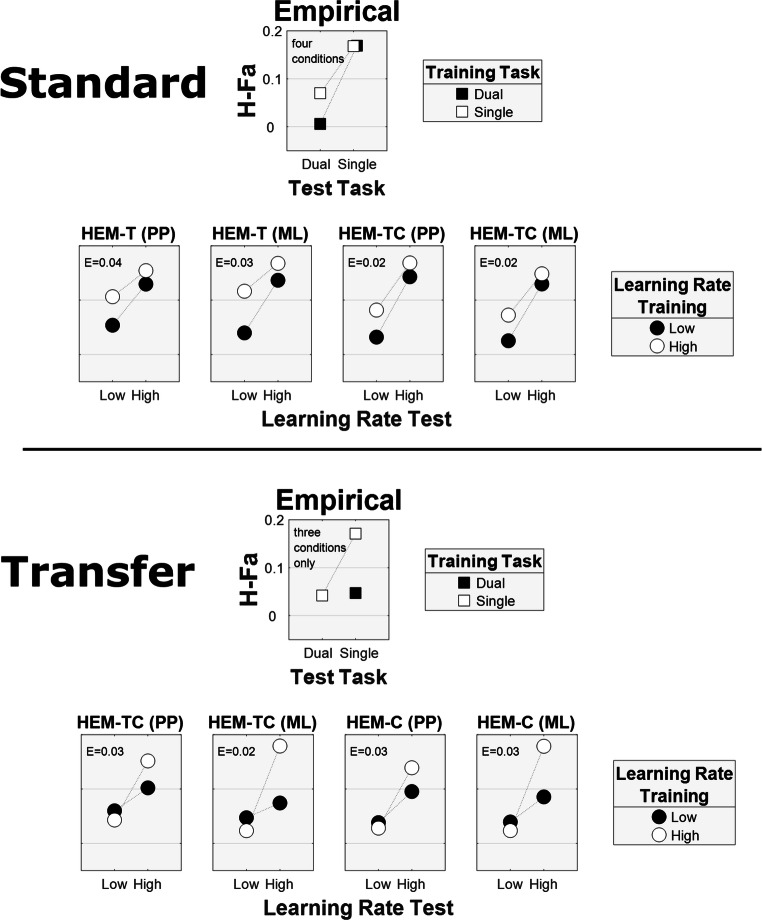
Posterior predictive checks for standard and transfer artificial grammar learning (AGL). The empirical plots are the results from Hendricks et al. (2013). The other plots are model results for different models (HEM-T, HEMT-TC, or HEM-C). The empirical plots illustrate combinations of single and dual training and test tasks. The model panels illustrate combinations of low and high learning rates at training and test. The vertical axis is *H* - *Fa* = Hits minus False alarms. *E* = root mean square error. The model results are based either on the maximum likelihood (*ML*) parameter estimates or on sampling from the posterior distributions (posterior predictive, PP) of model parameters. Each mean (circles) in the model plots is based on 100 iterations of 20 participants. Note that for transfer AGL the empirical data only involve three conditions, but the simulations include all four combinations. The scale is the same in all plots

Figure 2 shows the results of posterior predictive model checking, both for the posterior distribution of parameters (equivalent to the likelihood function for uniform priors) and for the maximum likelihood parameters The model plots show the mean model output with respect to posterior or maximum likelihood parameters and the empirical plots show the observed means from Hendricks et al. (2013).

First, consider the results for standard AGL (top part of Fig. 2). The HEM-TC model, which was supported over the HEM-T model by the Bayes factor, provides a good account of the data. The empirical data for standard AGL may invite the interpretation that learning is automatic, because a dual task at training has little effect on performance. However, the HEM-TC model accounts for this result by learning at test. The HEM-C model, which does not learn at test, could not account for the results. According to the test learning models, this pattern occurs because of the differential similarity constraints embodied in the training and test items. Simply learning at test is enough in this case. The empirical data also show that a dual task at test for standard AGL disrupts performance. According to the test learning models, this occurs because test learning is disrupted. Simply learning at training is not enough in this case.

The bottom part of Fig. 2 shows the results for transfer AGL. Although the model outcomes and the empirical data are not identical, the model outcomes capture the basic pattern of the empirical data, namely a detrimental effect of a dual task on classification regardless of when the dual task is realized (training or testing). The pattern observed by Hendricks et al. (2013) during transfer is that a dual task had the same detrimental effect on classification regardless of when the dual task was realized (during training or during testing). The current modeling framework invites two interpretations of this outcome, both of which stand as alternatives to that offered by Hendricks et al. (2013).

The first possibility is that both standard and transfer AGL involves test learning. The HEM-TC model captures the empirical basic data patterns in both cases. The second possibility is that standard AGL, but not transfer AGL, involves test learning. Of the tested models, only the ones involving test learning capture the results for standard AGL, but for transfer AGL HEM-C also captures the basic pattern. Although this second possibility may seem contrived at first sight, it can be theoretically motivated. Standard AGL experiments naturally invite test learning, because the training and test items are similar to each other and instantiated by the same surface features. Transfer experiments on the other hand, by their very nature, involve very salient cues signaling abrupt change when the surface features suddenly are different. This could have the effect of eliminating test learning under the impression that it is no longer relevant. In this case, the boundary between the training and test phases is more clearly delineated. In contrast to this idea, it has been suggested that transfer conditions in particular involve test learning, because even participants in untrained control groups have been observed to perform above chance (Redington & Chater, 1996). However, untrained control groups have no choice but to engage in test learning. Observing that untrained transfer controls perform above chance does not mean that trained transfer participants engage in test learning. The untrained group does not experience the change cues signaling a strong boundary between training and test, but the trained group does.

For current purposes it matters less which of the presented memory models accounts best for performance. What matters most is that they collectively provide a viable alternative that is not ruled out by the data. It could be that standard AGL is based on familiarity-based memory of the kind studied here, but that transfer AGL is based on different processes involving different kinds of mapping mechanisms (Dienes, Altmann, & Gao, 1999; Redington & Chater, 1996) that would be more sensitive to dual tasks. One challenge with finding support for multiple processes is to show that these multiple-process models can account for performance. However, an additional and often overlooked challenge is to rule out simpler models that do not involve separate processes. The extended HEM models presented here provide a demonstration of a set of alternative models not ruled out by data purported in Hendricks et al. (2013) to provide evidence for automatic versus intentional processes. Future work may consider contrasting different types of test learning models (cf. Rohrmeier & Cross, 2014), including models with multiple learning processes.

The history of implicit learning research is rich with examples of development and testing of computational models, but most of these simply implement test processes as application of acquired knowledge. The test learning mechanism implemented in the current HEM models goes beyond simple application of knowledge in the test phase. Nevertheless, the current test learning mechanisms are only simplified approximations in order to model these particular results. One important question concerns how to model more complicated forms of test learning. For example, Whittlesea, Brooks, and Westcott (1994) found that test phase manipulations affected the specificity of applied knowledge during the test phase. It seems plausible that such manipulations will affect not only how knowledge is applied, but also what is learnt during the test phase. As in the current work, implementing such processes in computational models may help shed light on the processes necessary to explain performance.

## References

[CR1] Beesley T, Wills AJ, le Pelley ME (2010). Syntactic transfer in artificial grammar learning. Psychonomic Bulletin and Review.

[CR2] Brooks LR, Vokey JR (1991). Abstract analogies and abstracted grammars: Comments on Reber (1989) and Mathews et al. (1989). Journal of Experimental Psychology: General.

[CR3] Curtis ET, Jamieson RK (2019). Computational and empirical simulations of selective memory impairments: Converging evidence for a single-system account of memory dissociations. Quarterly Journal of Experimental Psychology.

[CR4] Dienes Z, Altmann GTM, Gao S-J (1999). Mapping across domains without feedback: A neural network model of transfer of implicit knowledge. Cognitive Science.

[CR5] Dunn JC, Kirsner K (2003). What can we infer from double dissociations?. Cortex.

[CR6] Hasher L, Zacks RT (1979). Automatic and effortful processes in memory. Journal of Experimental Psychology: General.

[CR7] Hendricks MA, Conway CM, Kellogg RT (2013). Using Dual-Task Methodology to Dissociate Automatic From Nonautomatic Processes Involved in Artificial Grammar Learning. Journal of Experimental Psychology: Learning, Memory, and Cognition.

[CR8] Higham PA, Vokey JR, Pritchard JL (2000). Beyond dissociation logic: Evidence for controlled and automatic influences in artificial grammar learning. Journal of Experimental Psychology: General.

[CR9] Hintzman DL (1984). MINERVA 2: A simulation model of human memory. Behavior Research Methods, Instruments and Computers.

[CR10] Jamieson RK, Hauri BR (2012). An exemplar model of performance in the artificial grammar task: Holographic representation. Canadian Journal of Experimental Psychology.

[CR11] Jamieson RK, Holmes S, Mewhort DJK (2010). Global similarity predicts dissociation of classification and recognition: Evidence questioning the implicit/explicit learning distinction in amnesia. Journal of Experimental Psychology: Learning, Memory, and Cognition.

[CR12] Jamieson RK, Mewhort DJK (2011). Grammaticality is inferred from global similarity: A reply to Kinder (2010). Quarterly Journal of Experimental Psychology.

[CR13] Jamieson RK, Vokey JR, Mewhort DJK (2017). Implicit learning is order dependent. Psychological Research.

[CR14] Kinder A, Shanks DR (2001). Amnesia and the declarative/nondeclarative distinction: A recurrent network model of classification, recognition, and repetition priming. Journal of Cognitive Neuroscience.

[CR15] Kinder A, Shanks DR (2003). Neuropsychological dissociations between priming and recognition: A single-system connectionist account. Psychological Review.

[CR16] Lotz A, Kinder A (2006). Transfer in artificial grammar learning: The role of repetition information. Journal of Experimental Psychology: Learning, Memory, & Cognition.

[CR17] Nosofsky RM, Zaki SR (1998). Dissociations between categorization and recognition in amnesic and normal individuals: An exemplar-based interpretation. Psychological Science.

[CR18] Palmeri TJ, Flanery MA (1999). Learning about categories in the absence of training: Profound amnesia and the relationship between perceptual categorization and recognition memory. Psychological Science.

[CR19] Perruchet P, Pacteau C (1990). Synthetic grammar learning: implicit rule abstraction or explicit fragmentary knowledge?. Journal of Experimental Psychology: General.

[CR20] Reber, R., & Perruchet, P. (2003). The use of control groups in artificial grammar learning. *Quarterly Journal of Experimental Psychology, 56A*, 97-115. 10.1080/02724980244000297 12587897

[CR21] Redington M, Chater N (1996). Transfer in artificial grammar learning: A reevaluation. Journal of Experimental Psychology: General.

[CR22] Roediger HL, Smith MA (2012). The “pure-study” learning curve: The learning curve without cumulative testing. Memory and Cognition.

[CR23] Rohrmeier MA, Cross I (2014). Modelling unsupervised online-learning of artificial grammars: Linking implicit and statistical learning. Consciousness and Cognition.

[CR24] Servan-Schreiber E, Anderson JR (1990). Learning artificial grammars with competitive chunking. Journal of Experimental Psychology: Learning, Memory, and Cognition.

[CR25] Tunney RJ, Shanks DR (2003). Does opposition logic provide evidence for conscious and unconscious processes in artificial grammar learning?. Consciousness and Cognition.

[CR26] Vokey JR, Higham PA (2004). Opposition logic and neural network models in artificial grammar learning. Consciousness and Cognition.

[CR27] Whittlesea BWA, Brooks LR, Westcott C (1994). After the learning is over: Factors controlling the selective application of general and particular knowledge. Journal of Experimental Psychology: Learning, Memory, and Cognition.

[CR28] Zaki SR, Nosofsky RM (2007). A high-distortion enhancement effect in the prototype-learning paradigm: Dramatic effects of category learning during test. Memory and Cognition.

